# Loneliness, depression and sociability in old age

**DOI:** 10.4103/0972-6748.57861

**Published:** 2009

**Authors:** Archana Singh, Nishi Misra

**Affiliations:** Defence Institute of Psychological Research, Lucknow Road, Timarpur, Delhi - 110 054, India; 1 Scientist ‘C’, DIPR, DRDO, Ministry of Defence, Lucknow Road, Timarpur, Delhi - 110 054, India

**Keywords:** Depression, Loneliness, Old age, Sociability

## Abstract

**Background:**

The elderly population is large in general and growing due to advancement of health care education. These people are faced with numerous physical, psychological and social role changes that challenge their sense of self and capacity to live happily. Many people experience loneliness and depression in old age, either as a result of living alone or due to lack of close family ties and reduced connections with their culture of origin, which results in an inability to actively participate in the community activities. With advancing age, it is inevitable that people lose connection with their friendship networks and that they find it more difficult to initiate new friendships and to belong to new networks. The present study was conducted to investigate the relationships among depression, loneliness and sociability in elderly people.

**Materials and Methods:**

This study was carried out on 55 elderly people (both men and women). The tools used were Beck Depression Inventory, UCLA Loneliness Scale and Sociability Scale by Eysenck.

**Results:**

Results revealed a significant relationship between depression and loneliness.

**Conclusion:**

Most of the elderly people were found to be average in the dimension of sociability and preferred remaining engaged in social interactions. The implications of the study are discussed in the article.

Aging is a series of processes that begin with life and continue throughout the life cycle. It represents the closing period in the lifespan, a time when the individual looks back on life, lives on past accomplishments and begins to finish off his life course. Adjusting to the changes that accompany old age requires that an individual is flexible and develops new coping skills to adapt to the changes that are common to this time in their lives (Warnick, 1995).

The definition of ‘health’ with regard to old age is a subject of debate. There is consensus that health in old age cannot meaningfully be defined as the absence of disease because the prevalence of diagnosable disorders in elderly populations is high. Instead, health is considered to be multifaceted: The diagnosis of disease should be complemented by assessment of discomfort associated with symptoms (e.g., pain), life threat, treatment consequences (e.g., side effects of medication), functional capacity and subjective health evaluations (Borchelt *et al*., 1999). Furthermore, Rowe & Khan (1987) suggested that the health of subgroups of older adults be defined in terms of their status relative to age and cohort norms.

There is a growing body of evidence that suggests that psychological and sociological factors have a significant influence on how well individuals age. Aging research has demonstrated a positive correlation of someone’s religious beliefs, social relationships, perceived health, self-efficacy, socioeconomic status and coping skills, among others, with their ability to age more successfully.

Depression or the occurrence of depressive symptomatology is a prominent condition amongst older people, with a significant impact on the well-being and quality of life. Many studies have demonstrated that the prevalence of depressive symptoms increases with age (Kennedy, 1996). Depressive symptoms not only have an important place as indicators of psychological well-being but are also recognized as significant predictors of functional health and longevity. Longitudinal studies demonstrate that increased depressive symptoms are significantly associated with increased difficulties with activities of daily living (Penninx *et al*., 1998). Community-based data indicate that older persons with major depressive disorders are at increased risk of mortality (Bruce, 1994). There are also studies that suggest that depressive disorders may be associated with a reduction in cognitive functions (Speck *et al*., 1995).

Though the belief persists that depression is synonymous with aging and that depression is in fact inevitable, there has been recent research which dispels this faulty notion. Depression has a causal link to numerous social, physical and psychological problems. These difficulties often emerge in older adulthood, increasing the likelihood of depression; yet depression is not a normal consequence of these problems. Studies have found that age isn’t always significantly related to level of depression, and that the oldest of olds may even have better coping skills to deal with depression, making depressive symptoms more common but not as severe as in younger populations.

When the onset of depression first occurs in earlier life, it is more likely that there are genetic, personality and life experience factors that have contributed to the depression. Depression that first develops in later life is more likely to bear some relationship to physical health problems. An older person in good physical health has a relatively low risk of depression. Physical health is indeed the major cause of depression in late life. There are many reasons for this, which include the psychological effects of living with an illness and disability, the effects of chronic pain; the biological effects of some conditions and medications that can cause depression through direct effects on the brain; and the social restrictions that some illnesses place upon older people’s life style resulting in isolation and loneliness.

There are strong indications that depression substantially increases the risk of death in adults, mostly by unnatural causes and cardiovascular disease (Wulsin *et al*., 1999). Some population-based studies did find that this independent relationship does exist in later life, while others did not.

Loneliness is a subjective, negative feeling related to the person’s own experience of deficient social relations. The determinants of loneliness are most often defined on the basis of 2 causal models. The first model examines the *external factors*, which are absent in the social network, as the root of the loneliness; while the second explanatory model refers to the *internal factors*, such as personality and psychological factors.

Loneliness may lead to serious health-related consequences. It is one of the 3 main factors leading to depression (Green *et al*., 1992), and an important cause of suicide and suicide attempts. A study carried out by Hansson *et al*. (1987) revealed that loneliness was related to poor psychological adjustment, dissatisfaction with family and social relationships.

As people grow old, the likelihood of experiencing age-related losses increases. Such losses may impede the maintenance or acquisition of desired relationships, resulting in a higher incidence of loneliness. Many people experience loneliness either as a result of living alone, a lack of close family ties, reduced connections with their culture of origin or an inability to actively participate in the local community activities. When this occurs in combination with physical disablement, demoralization and depression are common accompaniments. The negative effect of loneliness on health in old age has been reported by researchers (Heikkinen *et al*., 1995). The death of spouse and friends and social disengagement after leaving work or a familiar neighborhood are some of the ubiquitous life-changing events contributing to loneliness in older people. Those in the oldest age cohort are most likely to report the highest rates of loneliness, reflecting their increased probability of such losses.

A study by Max *et al*. (2005) revealed that the presence of perceived loneliness contributed strongly to the effect of depression on mortality. Thus, in the oldest old, depression is associated with mortality only when feelings of loneliness are present. Depression is a problem that often accompanies loneliness. In many cases, depressive symptoms such as withdrawal, anxiety, lack of motivation and sadness mimic and mask the symptoms of loneliness.

## Sociability and old age

Sociability plays an important role in protecting people from the experience of psychological distress and in enhancing well-being. George (1996) summarized some of the empirically well-supported effects of social factors on depressive symptoms in later life, and reported that increasing age, minority racial or ethnic status, lower socioeconomic status and reduced quantity or quality of social relations are all associated with increased depressive symptom levels. Social isolation is a major risk factor for functional difficulties in older persons. Loss of important relationships can lead to feelings of emptiness and depression. “Persons involved with a positive relationship tend to be less affected by everyday problems and to have a greater sense of control and independence. Those without relationships often become isolated, ignored, and depressed. Those caught in poor relationships tend to develop and maintain negative perceptions of self, find life less satisfying and often lack the motivation to change” (Hanson & Carpenter, 1994).

Having few social contacts or living alone does not assure a state of loneliness (Mullins, Johnson, & Anderson, 1987). In fact, for elderly people the time spent with family may be less enjoyable than a visit to a neighbor or someone of their age group. This can be attributed to the fact that relationships with family tend to be obligatory whereas those with friends are a matter of choice. This further emphasizes the need for a perceived internal locus of control over social interaction as a means of alleviating loneliness.

Posner (1995) points out that older people tend to make friendships predominantly with those within the same age cohort. Thus with advancing age, it is inevitable that people lose their friendship networks and that they find it more difficult to initiate new friendships and to belong to new networks. However, those with more physical, material and intellectual resources also have more social “capital,” which allows them to continue to seek out new relationships and forms of social involvement.

The number of older people is increasing throughout the world. As individuals grow older, they are faced with numerous physical, psychological and social role changes that challenge their sense of self and capacity to live happily. Depression and loneliness are considered to be the major problems leading to impaired quality of life among elderly persons. At the same time, old age can also be an opportunity for making new friends, developing new interests, discovering fresh ways of service, spending more time in fellowship with God. It can be happy and winsome or empty and sad — depending largely on the faith and grace of the person involved. Therefore, the present study was undertaken with the main purpose of studying the relationships among depression, loneliness and sociability in a group of elderly people and also to determine gender differences with respect to the above relationships of variables.

## Objectives of the study

- Examine the relationships among loneliness, depression and sociability in elderly persons
- Study gender differences with respect to sociability, loneliness and depression among elderly persons

## Hypotheses

- There will be a positive relationship between loneliness and depression in old age.
- There will be a negative relationship between sociability and loneliness in old age.
- There will be a negative relationship between sociability and depression in elderly persons.
- There will be gender differences with respect to the variables sociability, loneliness and depression in elderly persons.

## MATERIALS AND METHODS

### Sample

The sample comprised of 55 elderly persons (35 men and 20 women) in the age group of 60-80 years. The mean age of the sample population was 67 years. The subjects for the sample were selected from the older adults of a Delhi-based region residing in the housing societies. These elderly persons were contacted personally, and the questionnaires were administered to them.

### Measures

#### The revised UCLA (University of California, Los Angeles) loneliness scale (Russell *et al*., 1980)

The UCLA Loneliness Scale includes 10 negatively worded and 10 positively worded items that have the highest correlations with a set of questions that are explicitly related with loneliness. The revised version of the scale has high discriminative validity. The revised loneliness scale also has a high internal consistency, with a coefficient alpha of 0.94.

#### Beck depression inventory (Beck *et al*., 1961)

The Beck Depression Inventory (BDI) is a 21-item self-report scale measuring supposed manifestations of depression. The internal consistency for the BDI ranges from 0.73 to 0.92, with a mean of 0.86. The BDI demonstrates high internal consistency, with alpha coefficients of 0.86 and 0.81 for psychiatric and nonpsychiatric populations, respectively. The scale has a split-half reliability coefficient of 0.93.

#### Sociability subscale of Eysenck personality profiler (Eysenck & Eysenck, 1975)

Eysenck Personality Profiler (EPP V6) is a multidimen sional modular personality inventory for 3 dimensions: Extroversion, emotionality (neuroticism) and adventurous ness (psychoticism). Each dimension has 7 subscales.

The sociability subscale of extroversion used in this study consists of 20 questions. The response category is either ‘yes’ or ‘no.’ There are 10 positive items and 10 negative items. The factorial validity of the EPP V6 holds across different cultures and age groups, with a high equivalent factor structure among these different samples.

### Procedure

Initially the participants were personally contacted and rapport was established with them. The participants completed the questionnaires given to them. Standard instructions were written on top of each questionnaire, and the participants were asked to rate themselves under the option they felt relevant to them. It was made clear to the participants that there were no right and wrong answers. If they had any difficulty, they were encouraged to ask questions. After finishing the entire set of questions, they were asked to return the questionnaires. The test administration took about 45minutes.

## RESULTS

Table 1 shown above reveals that there are no significant gender differences in elderly men and women with respect to loneliness and depression. Elderly men, however, were found to be more sociable as compared to elderly women.

**Table 1:** Means and standard deviations for gender differences for loneliness, depression and sociability

Variables	Men (*n* = 35) M (SD)	Women (*n* = 20) M (SD)	*T*	Sig (2 tailed)
Loneliness	47.43 (7.54)	45.75 (9.33)	0.73	0.47
Depression	18.74 (11.35)	22.6 (8.59)	–1.32	0.19
Sociability	8.91 (3.30)	7.20 (1.70)	2.16	0.036**

** *P* < .01

Table 2 shows a significant positive correlation between depression and loneliness, which is significant at the 0.01 level, i.e., there is an increase in the level of depression with an increase in loneliness among elderly men and women. A negative, though insignificant, relationship was found between sociability and loneliness. No significant relationship was found between sociability and depression.

**Table 2:** Correlations among loneliness, depression and sociability

Variables	Loneliness	Depression	Sociability
Loneliness	1.00		
Depression	0.528**	1.00	
Sociability	–0.010	0.032	1.00

** *P* < .01

Table 3 reveals that in the male elderly persons, a significant positive correlation was found between depression and loneliness. Sociability and loneliness were negatively correlated, though not significantly.

**Table 3:** Correlations among loneliness, depression and sociability (men)

Variables (Men)	Loneliness	Depression	Sociability
Loneliness	1.00		
Depression	0.557**	1.00	
Sociability	–0.118	0.050	1.00

** *P* < .01

Female elderly persons manifested a significant positive correlation between depression and loneliness, as can be seen in Table 4.

**Table 4:** Correlations among loneliness, depression and sociability (women)

Variables (Women)	Loneliness	Depression	Sociability
Loneliness	1.00		
Depression	0.602**	1.00	
Sociability	0.165	0.265	1.00

** *P* < .01

## DISCUSSION

The health and well-being of older adults is affected by the level of social activity and the mood states. Researchers have reported the negative effects of loneliness on health in old age (Heikkinen *et al*., 1995). Loneliness, coupled with other physical and mental problems, gives rise to feelings of depression in the elderly persons. Gender differences have been reported in the prevalence of health problems in elderly persons (Arber & Ginn, 1991). Results in Table 1 reveal that there are no significant gender differences in the elderly persons with respect to loneliness and depression, i.e., both the male and female elderly persons equally experience feelings of loneliness and depression. On the dimension of sociability, men were found to be more sociable as compared to their female counterparts. This may have been due to the fact that all the elderly men belonged to the working group, i.e., they were employed in government jobs before retirement and were less hesitant in socializing as compared to their female counterparts who were housewives and were spending their lives at home and finding pleasures by engaging in daily chores. Having both the intellectual and social resources allows elderly men to continue to seek out new relationships. Lack of significant gender differences on loneliness reflects the fact that since both the groups contained elderly married couples, with both partners being alive, the chances of their feeling lonely were low. Moreover, most of the couples were staying with their children and grandchildren, which did not allow them to stay lonely for long. Lack of significant gender differences on depression is contrary to the often held belief and research reports that elderly women are more prone to depression as compared to elderly men (Kessler *et al*., 1993). This result is not in line with what has been reported in literature. The findings of no significant gender differences with respect to depression may be attributed to the fact that all the women were nonworking ladies before they attained 60 years of age. Hence for them, the transition into old age was less associated with a change in life style associated with a break in ties with others or a sudden loss of power and status. The transition was very gradual, which prevented any abrupt change in mood states.

A positive correlation between loneliness and depression [Tables 2–4] is in accordance with the results obtained in literature with regard to both male and female elderly persons (Green *et al*., 1992). No significant relationship between loneliness and sociability [Table 2] reveals that despite being sociable, they experienced increased feelings of loneliness. Possible explanation for this may be that feeling lonely not only depends on the number of connections one has with others but also whether or not one is satisfied with his life style. An expressed dissatisfaction with available relationships is a more powerful indicator of loneliness (Revenson, 1982).

Lack of significant relationship between depression and sociability [Table 2] confirms the fact that depression is multicausal, i.e., it arises due to a host of factors, like declining health, significant loss due to death of a spouse, lack of social support. Also most of the elderly persons had moderate connections with their friends and family members, and they participated in daily activities.

On the basis of obtained findings, the following conclusions can be made:

1. A significant positive correlation exists between loneliness and depression.
2. No significant relationship was found between loneliness and sociability; depression and sociability.
3. Men are found to be more sociable than women.
4. A significant correlation was found between loneliness and depression in both men and women.

There were certain limitations in the study:

1. The sample size was restricted to few elderly persons. Hence in future, a similar study needs to be conducted on a larger section of the elderly population.
2. For determining gender differences, both male and female constituents of the sample should be equivalent in all respects.
3. Moreover, no formal diagnosis of depression was made in the sample used in the study. Self-report inventory was used for determining the level of depressive symptoms in the elderly persons.

Keeping in view the above limitations, longitudinal studies on a larger group of elderly men and women are needed in future.
